# Contemporary 0.55 T MRI to visualize interstitial lung disease – An exploratory study

**DOI:** 10.1016/j.ejro.2025.100720

**Published:** 2026-01-05

**Authors:** Nadine Bayerl, Claudius S. Mathy, Christina Bergmann, Tobias Bäuerle, Lisa C. Adams, Armin M. Nagel, Jörg H.W. Distler, Teresa Gerhalter, Michael Uder, Rafael Heiss, Stephan Ellmann

**Affiliations:** a Institute of Radiology, Friedrich-Alexander-Universität Erlangen-Nürnberg and University Hospital Erlangen, Maximiliansplatz 3, Erlangen 91054, Germany; b Department of Medicine 3 – Rheumatology and Immunology, Friedrich-Alexander-Universität Erlangen-Nürnberg and University Hospital Erlangen, Ulmenweg 18, Erlangen 91054, Germany; c Deutsches Zentrum für Immuntherapie (DZI), Friedrich-Alexander-Universität Erlangen-Nürnberg and University Hospital Erlangen, Ulmenweg 18, Erlangen 91054, Germany; d Department of Diagnostic and Interventional Radiology, University Medical Center of Johannes Gutenberg-University Mainz, Langenbeckstr. 1, Mainz 55131, Germany; e Department of Diagnostic and Interventional Radiology, Faculty of Medicine, Technical University of Munich, Ismaninger Str. 22, Munich 81675, Germany; f Department of Rheumatology, University Hospital Düsseldorf, Medical Faculty of Heinrich Heine University, Germany and Hiller Research Center, University Hospital Düsseldorf, Medical Faculty of Heinrich Heine University, Moorenstr. 5, Düsseldorf 40225, Germany; g Department of Neurology, Medical University of Graz, Auenbruggerplatz 22, Graz 8036, Austria; h RNZ. – Radiologisch-Nuklearmedizinisches Zentrum, Martin-Richter-Str. 43, Nürnberg 90489, Germany

**Keywords:** Magnetic resonance imaging, Computed tomography, X-Ray, Interstitial lung diseases, Scleroderma, Systemic, Diagnostic imaging

## Abstract

**Purpose:**

To evaluate the feasibility of contemporary 0.55 T MRI for visualizing interstitial lung disease (ILD) compared to high-resolution computed tomography (HRCT) in an exploratory first-experience study.

**Materials and methods:**

Thirty participants (mean age 60 ± 13 years; 13 females) with rheumatologic ILD underwent HRCT and 0.55 T MRI within 31 days. MRI protocols included proton-density-weighted turbo-spin-echo sequences (transverse) and T2-weighted short-tau inversion recovery sequences (coronal). Three blinded radiologists independently assessed overall disease extent, ground-glass opacity (GGO), reticulation, and emphysema using a semi-quantitative scoring system. Differences between modalities were tested using Wilcoxon signed-rank tests, and Bland-Altman analysis evaluated systematic bias.

**Results:**

Overall disease extent showed no statistically significant difference between low-field MRI and HRCT (median 22.5 % vs. 24.5 %), with excellent interobserver agreement (MRI ICC = 0.94; HRCT ICC = 0.97). MRI significantly overestimated GGO (13.1 % vs. 9.7 %) and underestimated reticulation (8.1 % vs. 11.4 %) compared to HRCT. Bland-Altman analysis confirmed no systematic bias for overall disease extent but consistent overestimation of GGO and underestimation of reticulation on MRI.

**Conclusions:**

Contemporary 0.55 T MRI showed no statistically significant difference in overall ILD extent compared to HRCT but tended to overestimate GGO and underestimate reticulation. Despite these limitations, 0.55 T MRI represents a promising candidate for future development as a radiation-free alternative for gross disease burden assessment in ILD, warranting further technical refinement before routine clinical use.

## Highlights

• Contemporary 0.55 T MRI assessed ILD extent comparably to HRCT.
• Ground-glass opacities were overestimated and reticulations underestimated on 0.55 T MRI compared to HRCT.
• 0.55 T MRI shows promise as a radiation-free ILD imaging option in the future.
• Technical advancements are needed before considering clinical use.

## 1 Introduction

Interstitial lung disease (ILD) is a diverse group of pulmonary disorders characterized by inflammation and fibrosis of the lung parenchyma, resulting in significant morbidity and mortality [1]. According to the Global Burden of Disease database 2019, ILD affects 57.62 cases per 100,000 population, with considerable healthcare burden including regular surveillance imaging [2]. The underlying causes can be inhalation of organic or inorganic dusts, post-infectious manifestations or autoimmune and collagen vascular diseases (syn. connective tissue diseases) such as systemic sclerosis (SSc), also referred to as connective tissue disease-associated interstitial lung disease (CTD-ILD). CTD-ILDs present a significant clinical challenge due to the complex pathogenesis and variable clinical manifestations [3]. High-resolution computed tomography (HRCT) has traditionally been used as the method of choice to detect, assess and follow-up ILD [4]. Patients with ILD thus undergo HRCT scans at intervals ranging from 3 to 6 months in patients with moderate-to-severe disease at baseline or with progressive disease to up to one year depending on the underlying disease, the ILD pattern and the extent, necessitating repeated radiation exposure over their lifetime [5].

HRCT allows detailed morphological visualization of typical patterns including usual interstitial pneumonia (UIP), which is characterized by honeycombing with a basal and peripheral predominance [6], [7]. The HRCT criteria for UIP pattern include reticular abnormalities with peripheral traction bronchiectasis and honeycombing, with or without ground glass opacities. In cases where reticular abnormalities and traction bronchiectasis with basal and subpleural predominance are observed without honeycombing, the pattern is classified as probable UIP. Non-specific interstitial pneumonia (NSIP) is characterized by ground-glass opacities (GGO), reticulations, and traction bronchiectasis. Unlike UIP, NSIP typically presents a more symmetrical distribution with relative sparing of the peripheral subpleural regions [6], [7].

Patients with ILD undergo HRCT scans at regular intervals up to once a year, with the frequency varying according to the stage of the disease [8], [9]. This monitoring is essential to detect progressive fibrosis at an early stage and to adjust therapy to prevent further irreversible lung damage. However, the repeated use of HRCT exposes patients to cumulative radiation, which raises concerns about long-term risks, particularly in young patients.

Recently, low-field magnetic resonance imaging (MRI) has emerged as a promising alternative for lung imaging, offering the possibility of visualizing lung pathologies without the risks associated with ionizing radiation [10], [11]. Advances in low-field MRI technology, including the application of specialized sequences such as proton density weighted (PDw) images with periodically rotated overlapping parallel lines with enhanced reconstruction (PROPELLER) [12], have improved the quality and diagnostic capabilities of pulmonary MRI [13], [14], [15]. While MRI as radiation-free modality offers numerous advantages including superior soft tissue contrast, it has not been widely used for lung imaging in the past due to limitations in its applicability. These include motion artifacts from breathing and heartbeat, low proton density in the lung compared to other tissues like the brain or liver, resulting in a low signal-to-noise ratio, and susceptibility differences at air-lung interfaces that create static local field gradients, leading to rapid signal decay [14], [16].

However, recent studies have demonstrated the feasibility of low-field MRI in visualizing pulmonary findings in patients with COVID-19 and other lung pathologies such as bronchiectases, mucus plugs, nodules, and lymphangioleiomyomatosis [10], [11], [13], [14], [17]. Despite these promising developments, low-field MRI systems such as 0.55 T - which inherently have fewer artifacts due to reduced magnetic susceptibility effects - remain unexplored for ILD assessment. To our knowledge, this represents the first study to systematically evaluate a modern 0.55 T MRI system for ILD imaging. The primary aim of this study was to evaluate the clinical performance of non-contrast-enhanced low-field MRI in visualizing ILD, focusing on quantifying the extent of overall ILD burden and its individual components (ground-glass opacity and reticulation), which are crucial for therapy decisions. We compared images from a modern 0.55 T low-field MRI system with those from HRCT, the current reference standard, to assess the diagnostic accuracy and potential benefits of MRI in visualizing and quantifying ILD.

## 2 Materials and methods

### 2.1 Study population

The prospective single-center trial was approved by the local ethics committee of the Friedrich-Alexander-Universität Erlangen-Nürnberg (approval number: 22-13-Bm). Before the study commenced, potential participants with ILD were identified by the rheumatology outpatient clinic of the University Hospital Erlangen and contacted by a physician who provided information about the study. If they expressed interest, the physician then screened them for any contraindications and asked for their consent to participate in the study.

**Fig. 1:**
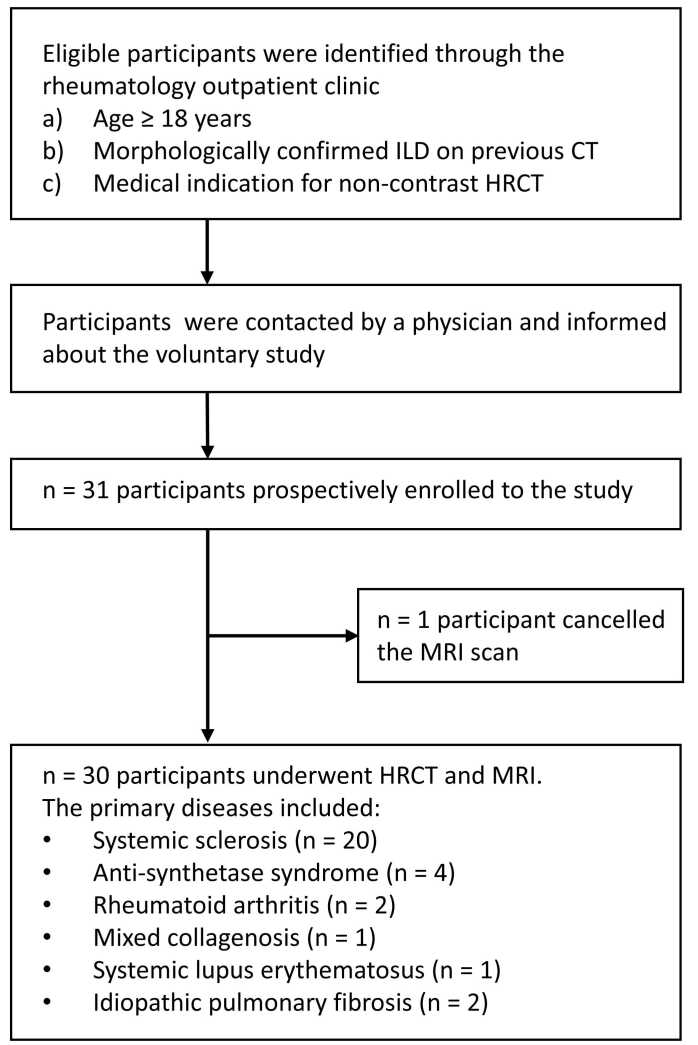
Flowchart of study population. ILD, interstitial lung disease; CT, computed tomography; HRCT, high-resolution computed tomography.

The eligibility criteria were defined as follows. Inclusion criteria: (a) age ≥ 18 years, (b) medical indication for chest HRCT, (c) morphologically confirmed fibrotic ILD, defined as presence of reticulation and/or honeycombing on a previous HRCT scan and its report in the radiology information system. Exclusion criteria included: (a) contraindications to MRI such as cardiac pacemaker or cochlear implant, pregnancy, claustrophobia, (b) poor general health. Between September 2022 and July 2023, the study enrolled 31 consecutive participants. One participant cancelled the MRI scan during the exam before the sequences were completed, leaving a total of n = 30 participants’ data sets available for image analysis. Among these patients, the underlying primary diseases were: SSc (n = 20), anti-synthetase syndrome (n = 4), rheumatoid arthritis (n = 2), idiopathic pulmonary fibrosis (n = 2), mixed connective tissue disease (n = 1), systemic lupus erythematosus (n = 1). Please refer to Fig. 1 for a summarized overview of the study population. All participants enrolled in the study gave written informed consent.

### 2.2 Imaging acquisition

Participants underwent both conventional HRCT and lung MRI. All HRCT and MRI scans were performed without the use of contrast agents.

### 2.3 Magnetic resonance imaging

Non-contrast MRI scans were performed on a 0.55 T scanner (MAGNETOM Free.Max; Siemens Healthineers AG, Forchheim, Germany). Participants were positioned in head-first supine position with arms down. Two-dimensional turbo spin-echo (TSE) sequences were acquired in transverse and coronal plane with free-breathing and gated image acquisition during expiration. In transverse plane, PDw sequences were acquired with PROPELLER readout. The T2 weighted (T2w) coronal sequences were acquired with a short-tau inversion recovery (STIR) preparation and a half-Fourier acquired single-shot turbo spin-echo (HASTE) readout. Please refer to Table 1 for a detailed overview of the acquisition parameters.

**Table 1:** Magnetic resonance imaging parameters.

	Acquisition parameters	
	PDw TSE	T2w TSE HASTE STIR
Orientation	transverse	coronal
Median TA, min:sec	06:44 (IQR: 05:52 – 07:58)	05:56 (IQR: 04:53 – 07:48)
TE/TR, ms	35/2000	77/2500
In-plane resolution, mm^2^	1.3 × 1.3	1.5 × 1.5
Matrix	304 × 304	272 × 272
Acceleration mode	GRAPPA	GRAPPA
Acceleration factor	2	2
Readout trajectory	PROPELLER	PROPELLER
Slice thickness, mm	6.0	6.0

PDw, proton density weighted; T2w, T2 weighted; TSE, turbo spin-echo; HASTE, half-fourier acquired single-shot turbo spin-echo; STIR, short-tau inversion recovery; TA, time of acquisition; TE, time of echo; TR, time of repetition; GRAPPA, generalized autocalibrating partial parallel acquisition; PROPELLER, periodically rotated overlapping parallel lines with enhanced reconstructions

For the visualization of morphological lung findings and for MRI evaluation, transverse plane PDw sequences were used. The coronal planes were used for anatomic correlation in accordance with the scoring system. Phased-array receiver coils were used for all examinations, including a 6-channel flex coil, a 9-channel spine coil and a 12-channel head and neck coil. The use of the latter as an additional array coil, provided a more homogeneous signal within upper thoracic aperture.

### 2.4 Computed tomography

Non-contrast HRCT scans of the chest were performed in inspiration with participants set up in head-first supine position with arms up. The scans were acquired on 128-slice CT scanners (SOMATOM go.Top, SOMATOM X.cite; Siemens Healthineers AG, Forchheim, Germany) and a 384-slice CT scanner (SOMATOM Force; Siemens Healthineers AG, Forchheim, Germany). Images were reconstructed in transverse plane with a slice thickness of 1.0 mm and an increment of 0.7 mm. For HRCT evaluation, DICOM images in the transverse plane of the lung window with standard HRCT parameters and a slice thickness of 1 mm were analyzed.

### 2.5 Image analysis

The scoring of the visualization of ILD was adapted from a clinically [18] and radiologically [19] established semi-quantitative scoring system to reflect for the clinical relevance of our study results. The DICOM images of the MRI and HRCT scans were analyzed separately by radiologists with 3, 5 and 10 years of radiological experience, respectively, who were selected based on their thoracic imaging expertise and availability for the study period on a diagnostic dual-monitor workstation using a dedicated PACS software (syngo.plaza; Siemens Healthineers AG, Forchheim, Germany). Each radiologist was blinded to the MRI and HRCT examinations and performed the assessment independently. Analysis was performed on transverse slices at five levels of the lung (1, origin of great vessels; 2, carina; 3, pulmonary venous confluence; 4, between 3 and 5; 5, 1 cm above the right hemidiaphragm) according to the previously established scoring system. Coronal planes on HRCT and MRI were used to correlate the localization of the five defined levels for scoring.

The following criteria were scored to the nearest 5 %: overall extent of interstitial lung disease, extent of GGO, extent of reticulation, and extent of emphysema. Overall disease extent was defined as the percentage of lung parenchyma affected by any ILD-related abnormality. As defined by the Fleischner Society [20], GGO was reported as increased lung attenuation without obscuration of underlying vessels, while reticulation was defined as a net of intersecting linear opacities. Emphysema was defined as areas of abnormally low attenuation due to enlarged air spaces related to the terminal bronchioles with destroyed alveolar walls. The extent of ground-glass opacity and reticulation is given as a relative proportion; the sum of both is thus 100 % by definition. While honeycombing was included in the assessment of overall disease extent, it was not evaluated as a separate pattern due to its relatively low prevalence in our cohort. Prior to the formal image assessment, all three radiologists underwent a consensus training session using representative ILD cases (both MRI and HRCT) to standardize the scoring methodology and ensure consistent application of the semi-quantitative criteria.

### 2.6 Statistical analysis

The ILD scoring was assessed for normal distribution using Shapiro-Wilk tests, which revealed significant deviations from normal distributions. Therefore, values for this scoring are presented as median and interquartile range (IQR), and nonparametric paired-Group Wilcoxon signed-rank tests were performed to evaluate the differences between the MRI and HRCT groups. Bland-Altman analysis was performed to determine and visualize the agreement between MRI and HRCT and whether there were systematic differences between the two modalities studied, with respect to the previously defined criteria included in the scoring system. For statistical analysis, Prism version 9.5.1. (GraphPad Software, LCC, Boston, Massachusetts; USA) was used. Statistical significance was considered for p-values < 0.05. Highly significant differences were assumed for p-values < 0.01. Interobserver agreement was interpreted by intraclass correlation coefficient (ICC; two-way mixed model, absolute agreement, multiple raters) using IBM SPSS Statistics version 28 (IBM, Armonk, New York; USA). ICC values < 0.5 were considered as poor agreement, values between 0.5 – 0.75 as moderate agreement, values between 0.75 – 0.9 as good agreement, and values > 0.90 as excellent agreement [21].

## 3 Results

### 3.1 Study population

A total of 30 participants with a mean age ± standard deviation (SD) of 60 ± 13 years, ranging from 39 to 86 years, including 17 males and 13 females, completed the study. Within a maximum of 31 (IQR = 0 – 7.25) days, participants underwent both conventional HRCT, and lung MRI. The study cohort consisted of 21 patients with an NSIP pattern (70 %), which is consistent with the high proportion of CTD-ILD cases in our sample. A definite UIP pattern was observed in 6 patients (20 %), while a probable UIP pattern was found in 3 patients (10 %).

### 3.2 Image analysis

**Fig. 2:**
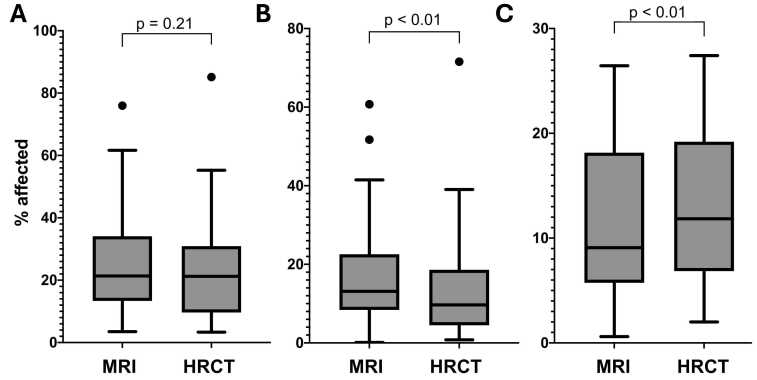
Boxplots comparing the overall disease extent as assessed with MRI and HRCT (A), the extent of ground-glass opacities (B), and the extent of reticulations (C). Boxplots follow the Tukey definition, with boxes ranging from the first to the third quartile, and the whiskers indicating the 1.5x interquartile range. Outliers are marked with dots. p-values from paired group Wilcoxon signed-rank tests are given above the plots. MRI, magnetic resonance imaging; HRCT, high-resolution computed tomography.

**Fig. 3:**
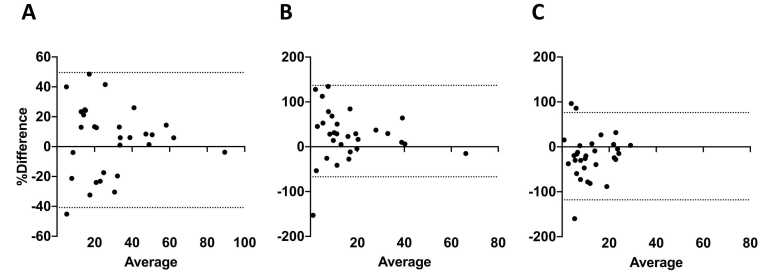
Bland-Altman plots to assess systematic biases of the assessments with MRI versus HRCT for overall disease extent (A), ground-glass opacities (B) and reticulation (C). The plots depict relative differences of the assessments against the average measurements, along with the 95 % confidence interval (dotted horizontal lines). Higher values on the y-axes result from a relatively higher parenchymal involvement in the MRI assessment compared to HRCT, as particularly obvious for the ground-glass opacities in subplot (B).

In the analysis of the overall disease extent of ILD, low-field MRI of the lung provided a comparable assessment to HRCT as illustrated in Fig. 2, Fig. 3 (MRI, median = 22.5 %, interquartile range IQR = 15 % – 42 %; HRCT, median = 24.5 %, IQR = 13 % – 36 %), with no significant differences between the two modalities (p = 0.21). Interobserver agreement in the assessment of overall disease extent was excellent for both MRI and HRCT (MRI, ICC = 0.94; HRCT, ICC = 0.97). The extent of GGO was significantly overestimated on MRI compared to HRCT (MRI, median = 13.1 %, IQR = 9 % – 23 %; HRCT, median = 9.7 %, IQR = 5 % – 19 %) with p = 0.0024. Interobserver agreement for assessment of the extent of GGO was good on MRI (ICC = 0.88) and excellent on HRCT (ICC = 0.94). The extent of reticulation was significantly underestimated on MRI compared to HRCT (MRI, median = 8.1 %, IQR = 6 % – 19 %; HRCT, median 11.4 %, IQR = 7 % – 20 %) with p = 0.0083. Interobserver agreement for assessment of the extent of reticulation was good on both MRI and HRCT (MRI, ICC = 0.8; HRCT, ICC = 0.85). Emphysema was notably rare in our cohort, with median values of 0 % (IQR = 0 % – 0.4 %) on MRI and 0 % (IQR = 0 % – 4.7 %) on HRCT, however, with MRI though significantly underestimating emphysema extent (p = 0.004). The interobserver agreement for assessment of the extent of emphysema was moderate on MRI (ICC = 0.71) and good on HRCT (ICC = 0.78). Bland-Altman analyses were performed to detect possible systematic biases (Fig. 3). While none of these biases were observed for the overall disease extent (Fig. 3A), the overestimation of GGO on MRI compared to HRCT and the underestimation of reticulation on MRI compared to HRCT were also revealed by the Bland-Altman plots (Fig. 3B and Fig. 3C, respectively).

### 3.3 Clinical cases

**Fig. 4:**
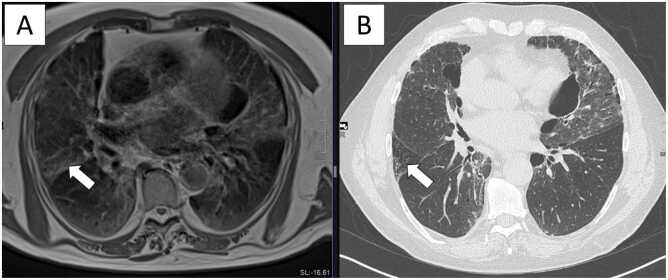
Clinical Case #1: 72-year-old male patient with rheumatoid arthritis-related interstitial lung disease. The depicted scoring level is level 3 (pulmonary venous confluence). The patient underwent (A) low-field MRI of the lung with a proton density weighted sequence and (B) HRCT of the chest on the same day. Overall disease extent was estimated almost equal on MRI and HRCT (MRI, median 48 %; HRCT, median 49 %) and included areas of honeycombing and ground-glass opacities as predominant patterns in this particular patient. The extent of ground-glass opacity was slightly overestimated (MRI, median 41.1 %; HRCT, median 36.5 %), and reticulation was underestimated on MRI compared to HRCT (MRI, median 8.1 %; HRCT, median 10.5 %). Particularly in the right lung, areas of reticulation visible on HRCT rather appear as ground-glass opacity on MRI (arrows).

**Fig. 5:**
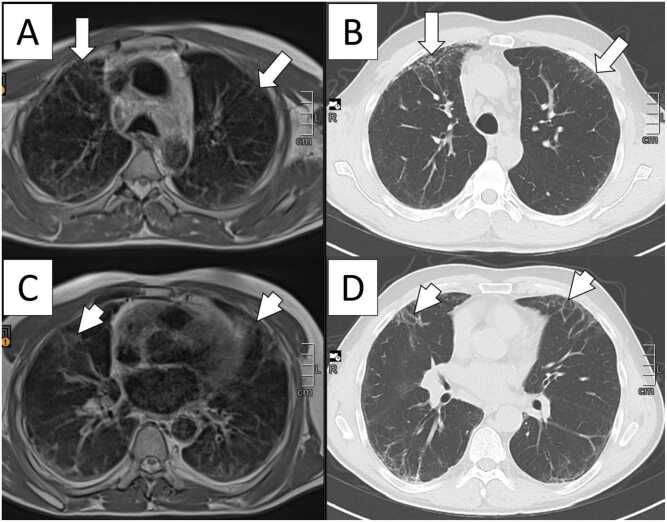
Clinical Case #2: 52-year-old male patient with systemic sclerosis-related interstitial lung disease with predominantly reticular pattern who underwent (A, C) low-field MRI of the lung with a proton density weighted sequence and (B, D) HRCT of the chest on the same day. The depicted scoring levels are level 2 (carina, A and B) and level 3 (pulmonary venous confluence, C and D). Overall disease extent was estimated almost equal (MRI, median 36 %; HRCT, median 33 %). The extent of ground-glass opacity was overestimated on MRI compared to HRCT (MRI, median 11.6 %; HRCT, median 6.0 %), whereas the extent of reticulation was underestimated on MRI (MRI, median 21.4 %; HRCT, median 27.0 %). At level 2 (A, B), reticulation in both anterior upper lobes can be depicted on HRCT as well as on MRI; however, it is less extensive on MRI (long arrows). At level 3 (C, D), reticulation on MRI becomes less clearly depictable and rather appears as a ground-glass opacity-like pattern (short arrows).

Fig. 4, Fig. 5 present Clinical Cases #1 and #2 with MRI and HRCT scans of two representative cases with ILD. In Clinical Case #1 (Fig. 4) overall disease extent was estimated almost equal on MRI and HRCT. In Clinical Case #2 (Fig. 5) overall disease extent was also rated quite similar on MRI and HRCT. In both cases, GGO tended to be overestimated on MRI compared to HRCT, and reticulations tended to be underestimated on MRI compared to HRCT, particularly visible in areas where reticulation on HRCT rather appears as GGO on MRI. Table 2 provides a detailed overview of the ratings.

**Table 2:** Median ratings of clinical cases #1 and #2 of overall disease extent, ground-glass opacities and reticulation.

	Ratings of Clinical Case #1		Ratings of Clinical Case #2	
	MRI	HRCT	MRI	HRCT
Overall Extent (median, %)	48	49	36	33
GGO (median, %)	41.1	36.5	11.6	6.0
Reticulation (median, %)	8.1	10.5	21.4	27.0

GGO, ground-glass opacities; MRI, magnetic resonance imaging; HRCT, computed tomography

## 4 Discussion

To the best of our knowledge, this work represents the first exploratory study evaluating the use of a modern 0.55 T MRI system for imaging ILD, a field traditionally dominated by HRCT scans due to their superior resolution and established diagnostic value. While HRCT remains the current reference standard for ILD imaging for staging and follow-up, its repeated use raises concerns due to cumulative radiation exposure over a lifetime, highlighting the importance of alternative imaging modalities to minimize patient risk in long-term disease management [4]. The 0.55 T MRI system represents an emerging technology specifically designed to increase accessibility of MRI, including in private practice settings. This field strength offers advantages such as reduced installation requirements and lower operating costs while maintaining diagnostic capability compared to higher field strengths. Moreover, it is less artifact prone on tissue-air surfaces such as the lung [22].

Our study evaluated the performance of low-field lung MRI in visualizing and quantifying lung fibrosis and its components: ground-glass opacity and reticulation pattern. Our data show that low-field MRI with its non-contrast sequences (PDw and T2w HASTE STIR) provides high agreement with HRCT in assessing the overall ILD extent with excellent interobserver agreement (MRI, ICC = 0.94; HRCT, ICC = 0.97). Since no significant differences were found between the two modalities in the assessment of overall ILD extent (p = 0.21), low-field MRI may represent a radiation-free option for visualizing ILD in the future. However, the study also highlighted the challenges in the MRI-based assessment of specific ILD components, and why this is not yet feasible for clinical implementation. Although PDw PROPELLER sequences in the transverse plane showed that affected lung parenchyma could be accurately quantified in terms of its overall extent using both low-field MRI and HRCT [17], reticulation was significantly underestimated on MRI compared to HRCT (p = 0.0083) and GGO was significantly overestimated (p = 0.0024). The observed discrepancy in GGO and reticulation quantification has multifactorial origins. Firstly, the lower spatial resolution of MRI (in-plane resolution approximately 1.6 × 1.6 mm) compared to HRCT (sub-millimeter resolution) limits the visualization of fine reticular structures, which are often less than 1 mm in thickness. Secondly, the substantially increased slice thickness of MRI (6 mm) compared to HRCT (1 mm) results in greater partial volume averaging, whereby fine reticulations within a voxel are averaged with adjacent ground-glass or normal parenchyma, making them less conspicuous. Future studies using different acquisition strategies (e.g., isotropic 3D sequences) might overcome this limitation. Thirdly, the inherent tissue contrast mechanisms differ between modalities: MRI signal intensity is determined by proton density and T1/T2 relaxation properties, which may be similarly altered in both inflammatory (GGO) and early fibrotic (fine reticulation) changes, potentially causing overlap in their MRI appearance. In contrast, HRCT depicts differences in physical density, allowing clearer distinction between these patterns. These technical limitations could impact clinical decisions, particularly in the grading of disease severity and progression, as accurate differentiation between potentially reversible inflammation (GGO) and irreversible fibrosis (reticulation) is crucial for therapeutic planning. Overestimation of GGO and underestimation of reticulation on MRI may lead to misinterpretation of the disease stage and suboptimal treatment strategies. Similar challenges have been observed in different studies, such as post-COVID lung assessments, with moderate to substantial intermodality agreement in the detection and characterization of GGO and reticulations on low-field MRI and HRCT [23]. Our findings on the overestimation of ground-glass opacity on MRI are, however, possibly influenced by the high prevalence of NSIP patterns in our cohort, which are characterized by extensive GGO. As an NSIP-pattern was observed in 70 % of our cases, this pattern-specific bias may have contributed to the discrepancy between MRI and HRCT in quantifying ground-glass changes. These observations highlight the need for future studies to include more diverse patterns, particularly fibrotic ILD subtypes, to evaluate how MRI performs across a wider range of ILD manifestations.

While subtle alterations such as GGO and reticulations seem to represent a particular challenge, pathologies with higher density like nodules and consolidations have been shown to be detectable in a more reliable way over different modalities [15], [24], [25].

The lower spatial resolution of MRI compared to HRCT may also impact the detection and characterization of honeycombing, potentially affecting pattern recognition and disease classification. Although honeycombing was not analyzed as a separate pattern in our study, its visualization on low-field MRI deserves specific attention in future research. The capacity to detect honeycombing reliably could be particularly relevant for identifying UIP pattern in various ILD subtypes, including SSc-ILD cases that develop UIP-like features [26], [27], [28].

While specialized sequences such as ultra-short echo time (UTE) imaging and higher field strengths (1.5 T) have shown promise in ILD assessment [26], [27], with some studies demonstrating agreement with HRCT in disease extent quantification, their clinical implementation remains limited. The Fleischner Society currently considers MRI use in ILD as investigational [28], though it supports lung MRI for other indications including cystic fibrosis and lung cancer staging. Low-field MRI systems (0.55 T), which inherently produce fewer susceptibility artifacts, remain underexplored for ILD despite potential advantages.

One reason for this may be that, although MRI has shown promise in detecting and categorizing ILD, the overall ILD extent has been inconsistently reported in studies comparing MRI and HRCT. While some studies, such as Landini et al., demonstrated agreement between 1.5 T MRI and HRCT in assessing ILD extent and stratification using advanced sequences such as T2w radial TSE and PDw UTE [29], others report systematic underestimation of ILD extent on 1.5 T MRI compared to HRCT [27]. Despite these advancements, low-field MRI systems such as 0.55 T, which inherently have fewer artifacts due to reduced magnetic susceptibility effects [22], are still underexplored. This leaves a gap in understanding their potential clinical utility for robust ILD assessment, though robust assessment of the overall ILD extent remains essential for therapeutic decisions [18].

Our decision to conduct this feasibility study in a predominantly CTD-ILD cohort was based on several practical and methodological considerations. First, these patients are followed at regular intervals in our rheumatology center, requiring frequent imaging for disease monitoring. This makes them ideal candidates for evaluating radiation-free alternatives to HRCT. Second, CTD-ILD patients, particularly those with SSc-ILD, often require lifelong monitoring starting at a younger age, making them a population that would especially benefit from reduced radiation exposure. However, we acknowledge that this population choice introduces important limitations. The predominance of NSIP pattern in CTD-ILD, characterized by extensive GGO, likely influenced our finding of GGO overestimation on MRI. This pattern-specific bias may not be equally applicable to other ILD subtypes where different patterns predominate. Future studies should include a more balanced representation of ILD patterns to better understand how MRI performance varies across different radiological patterns. From a clinical perspective, these findings suggest that whilst 0.55 T MRI cannot currently substitute for HRCT in distinguishing disease patterns critical for diagnosis and treatment planning, it may serve as a radiation-sparing alternative for monitoring overall disease burden in patients requiring frequent follow-up imaging. This could be particularly relevant for young patients with CTD-ILD who face decades of disease surveillance.

Our study focused exclusively on patients with fibrotic ILD, which allowed us to evaluate MRI performance in detecting established fibrotic changes. While this provided valuable insights into MRI's capability to assess fibrotic features, it also means our findings may not be directly applicable to early or predominantly inflammatory ILD cases. The assessment of early, non-fibrotic ILD changes on low-field MRI remains an important area for future research.

The very low prevalence of emphysema in our cohort (median 0 % on both modalities) is consistent with our predominantly CTD-ILD population, particularly SSc-ILD, where emphysema is uncommonly found. While our study demonstrates that MRI tends to underestimate emphysema compared to HRCT, the clinical significance of this finding is limited by the minimal emphysema present in our patient population. Future studies specifically including patients with combined pulmonary fibrosis and emphysema would be valuable to assess MRI's capability in detecting and quantifying emphysema. Further limitations of this study include the small sample size and its single-center design. While a sample size of 30 participants was sufficient to demonstrate the feasibility of low-field MRI in ILD assessment, larger multi-center studies with more diverse ILD subtypes would be valuable to validate these findings across different ILD patterns. The predominance of CTD-ILD cases in our cohort, while reflecting our center's rheumatology focus, may limit generalizability to other ILD subtypes. Furthermore, grading was performed semi-quantitatively by three readers from the same institute, which is a potential confounding factor, as the raters received identical training and may tend to interpret certain findings in a similar way. Second, the interval between HRCT and MRI was up to 31 days. Because of the slow progression of ILD over months to years [30], [31], we accepted this limitation to be able to recruit a sufficient number of patients from a relatively rare patient cohort of rheumatological disease and to provide a first experience of the performance of modern 0.55 T MRI compared with HRCT as the reference standard. To avoid misleading results, patients who did not undergo MRI on the same day as HRCT were asked beforehand whether they suffered an infection or exacerbation in the time since HRCT. MRI and HRCT scans can show differences due to respiratory movement; HRCT scans are acquired during inspiration, MRI scans during expiration. This discrepancy, together with the longer scan times required for MRI, can lead to dystelectasis in the lower pulmonary lobes, potentially obscuring findings in the posterior regions of the lower thorax.

Regarding the practical feasibility of transitioning from HRCT to MRI for ILD surveillance, several healthcare system considerations merit discussion. Whilst MRI infrastructure is less widely available than CT in many healthcare settings, low-field systems such as 0.55 T offer advantages in terms of reduced installation requirements, lower operating costs, and compatibility with standard clinical environments including those with limited space or power supply [10], [11], [15]. The absence of ionizing radiation makes MRI particularly valuable for younger patients and those requiring lifelong monitoring, potentially offsetting higher per-scan costs through elimination of radiation-associated risks. However, current limitations in pattern-specific assessment (GGO vs. reticulation) mean that MRI cannot yet replace HRCT for initial diagnosis and detailed disease characterization. A hybrid approach - using HRCT for diagnostic assessment and baseline characterization, with MRI for subsequent surveillance of overall disease burden - may represent a pragmatic pathway forward, particularly in specialized centers with appropriate MRI expertise.

In conclusion, non-contrast low-field MRI shows promise as a radiation-free alternative to HRCT for assessing overall ILD disease burden but requires further technical advancements, particularly in distinguishing ground-glass opacity from reticulation, before clinical implementation for comprehensive ILD assessment. Whilst it cannot currently replace HRCT for pattern-specific diagnosis, 0.55 T MRI represents a promising candidate for disease burden monitoring in patients requiring frequent surveillance imaging, warranting further investigation in larger, multi-center studies with diverse ILD subtypes.

## CRediT authorship contribution statement

**Rafael Heiss:** Writing – review & editing, Supervision, Software, Resources, Project administration. **Michael Uder:** Writing – review & editing, Supervision, Software, Resources, Project administration. **Nadine Bayerl:** Writing – review & editing, Writing – original draft, Visualization, Validation, Project administration, Methodology, Investigation, Funding acquisition, Formal analysis, Data curation, Conceptualization. **Stephan Ellmann:** Writing – review & editing, Visualization, Validation, Investigation, Formal analysis. **Christina Bergmann:** Writing – review & editing, Resources, Project administration. **Claudius S. Mathy:** Writing – review & editing, Investigation. **Lisa C. Adams:** Writing – review & editing, Supervision, Project administration, Investigation. **Tobias Bäuerle:** Writing – review & editing, Supervision, Software, Resources, Project administration. **Jörg H.W. Distler:** Writing – review & editing, Resources, Project administration. **Armin M. Nagel:** Writing – review & editing, Supervision, Software, Resources, Project administration. **Teresa Gerhalter:** Writing – review & editing, Software, Resources, Project administration.

## Ethical statement

This prospective single-center study complies with the Declaration of Helsinki. Informed consent was obtained from all participants. The local ethics committee of the Friedrich-Alexander-Universität Erlangen-Nürnberg approved the study (approval number: 22–13-Bm).

## Funding

NB is supported by the Interdisciplinary Center for Clinical Research (IZKF) of the Medical Faculty of the Friedrich-Alexander-Universität Erlangen-Nürnberg (Clinician Scientist Program, Laboratory Rotation Program), and by the Deutsche Forschungsgemeinschaft (DFG, German Research Foundation) – 493624887 (Clinician Scientist Program NOTICE). The remaining authors did not receive support from any organization for the submitted work.

## Declaration of Competing Interest

CSM, AMN, MU, RH are part of the speakers’ bureau of Siemens Healthineers. For the remaining authors no conflicts of interest and no funding sources were declared for this work.
